# Predicting disease progression from short biomarker series using expert advice algorithm

**DOI:** 10.1038/srep08953

**Published:** 2015-05-20

**Authors:** Kai Morino, Yoshito Hirata, Ryota Tomioka, Hisashi Kashima, Kenji Yamanishi, Norihiro Hayashi, Shin Egawa, Kazuyuki Aihara

**Affiliations:** 1 Graduate School of Information Science and Technology, The University of Tokyo, Tokyo 113-8656, Japan; 2 Institute of Industrial Science, The University of Tokyo, Tokyo 153-8505, Japan; 3 Toyota Technological Institute at Chicago, Chicago, Illinois 60637, USA; 4 Graduate School of Informatics, Kyoto University, Kyoto 606-8501, Japan; 5 CREST, JST, Honcho, Kawaguchi, Saitama 332-0012, Japan; 6 Department of Urology, Jikei University School of Medicine, Tokyo 105-8461, Japan

## Abstract

Well-trained clinicians may be able to provide diagnosis and prognosis from very short biomarker series using information and experience gained from previous patients. Although mathematical methods can potentially help clinicians to predict the progression of diseases, there is no method so far that estimates the patient state from very short time-series of a biomarker for making diagnosis and/or prognosis by employing the information of previous patients. Here, we propose a mathematical framework for integrating other patients' datasets to infer and predict the state of the disease in the current patient based on their short history. We extend a machine-learning framework of “prediction with expert advice” to deal with unstable dynamics. We construct this mathematical framework by combining expert advice with a mathematical model of prostate cancer. Our model predicted well the individual biomarker series of patients with prostate cancer that are used as clinical samples.

Mathematical models of diseases have been constructed to understand the mechanisms of diseases^1^,^2^,^3^,^4^,^5^,^6^,^7^, provide diagnosis and prognosis^8^,^9^,^10^, and determine treatment options^11^,^12^,^13^,^14^. When we focus on a clinical setting, it is crucial that we can estimate the state of a disease from short biomarker observations. Clinicians make such estimations using their experience with previous patients (see Fig. 1a). To the best of our knowledge, such estimations have not been realized mathematically thus far. If such mathematical estimation is possible, then we can optimize a treatment option in a personalized way. The difficulty of estimations stems not only from a lack of information, but also the instability of biomarkers' time-series, such as those for cancer volumes. Thus, our goal is to infer the state of a disease from both short, unstable time-series data of biomarkers obtained from a target patient and longer time-series data of biomarkers from previous patients who suffered from the same disease. We adopt the machine-learning framework of “online prediction”, which integrates “experts' advice”^15^ to make accurate predictions, where experts are short-term patterns of previous patients' histories, which are conformed to the target patient time-series.

A series of samples from a patient contains information on (often unstable) disease dynamics^8^,^10^ such as rapid increase. By considering the time series observed from the unstable dynamics, we may be able to better understand the current disease state. Employing past patients' time-series as experts and the target patient's time-series as observations, we can predict a time-series with the standard *expert advice* method^15^. However, this cannot be used directly, because we must deal with the unstable dynamics in which the value of a biomarker increases rapidly. In this paper, we propose an approach that couples an existing machine-learning technique with the instability possessed by the temporal disease datasets. Our method is based on the standard expert advice^15^, but deals with the instability of the underlying dynamics^8^,^10^ by integrating trajectories in a database with weights that increase exponentially in time.

## Results

### The proposed method: temporal expert advice (TEA)

**Figure 1:**
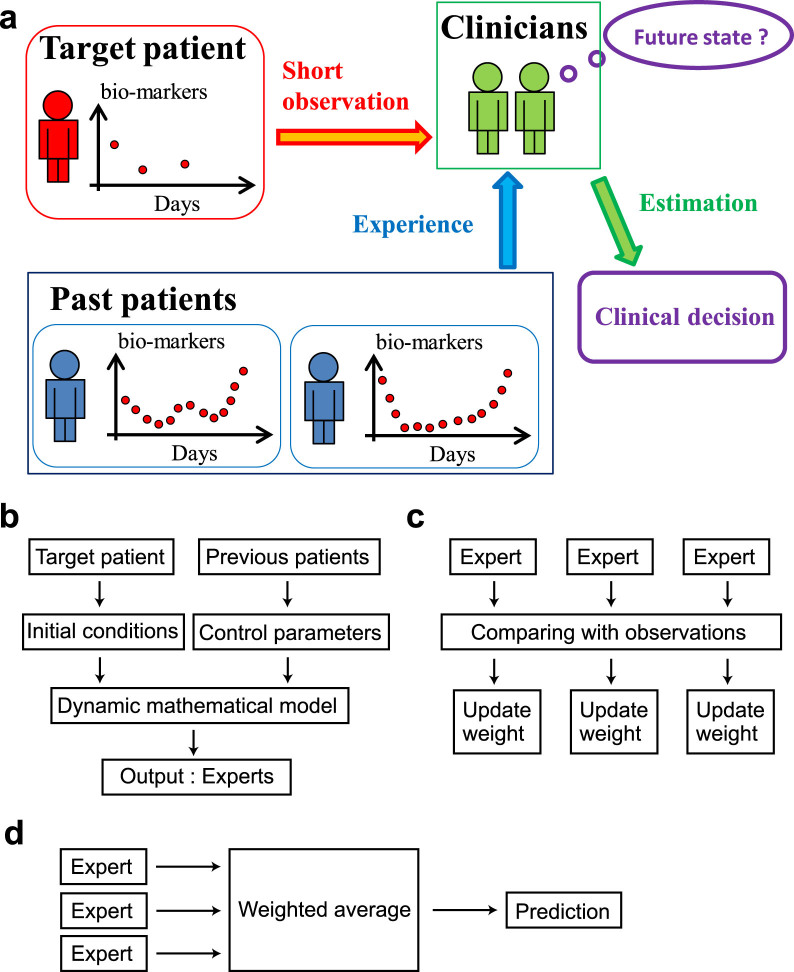
Schematic illustration of the estimation from short observations of biomarkers. (a) The prediction of clinicians. (b) The first step of TEA. (c) The second step of TEA. (d) The third step of TEA.

We extend the standard expert advice method^15^ to one that emphasizes near-past information. This *temporal expert advice*, or the TEA algorithm, consists of three steps (see Fig. 1b–d). TEA uses a collection of time-series, which we call experts, and weights each expert based on its agreement with the target time-series. The algorithm outputs a prediction by combining these experts.

The first step constructs an expert of a target system. There are two options. The first option is to use long time-series observed in the past as they are. We construct experts by simply inserting previous parts of the time-series or the datasets of previous patients. Let *x_j_*_,*l*_ be the *l*th point of the *j*th time-series in a database (*j* = 1, 2, …, *J*, *l* = 1, 2, …, *L*) and *f_i_*_,*t*_ be the *i*th expert's advice at time *t*. Numbers *J* and *L* are the number of time-series and the number of points in each time-series, respectively; We assume that the lengths of the time-series are equal, but it is easy to extend to cases of different lengths. Let *P* be the number of points related to each expert. Then, we can define an expert *f*_(*L*−*P*+1)(*j*−1)+*i*,*k*_ = *x_j_*_,*i*+*k*−1_ for *i* = 1, 2, …, *L* − *P* + 1, *k* = 1, 2, …, *P*. The second option is roughly to fit a mathematical model that has a set of parameters to a very short time-series, obtain the initial conditions for each set of parameters, and prepare the set of experts with these parameters. The details of this second rough option are discussed after we introduce a mathematical model of prostate cancer in the later section.

In the second step, TEA weights the trajectories *f_i_*_,*t*_ in the database to generate an appropriate weighting for the most current state. Let *y_k_* denote the observation at time *k*, and let *l*(·,·) be a loss function. When we include the next point, the weights *w_i_*_,*t*_ are updated according to a formula obtained by modifying the standard expert advice^15^. To achieve this, we sum the loss at each time step with a coefficient as follows:
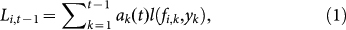

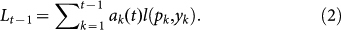
The modified form considers the instability of the underlying dynamics by introducing a coefficient *a_k_*(*t*), which measures the reliability of the prediction at, and increases exponentially with, time *k* such that *a_k_*(*t*) = *λ^k^*^−1^ or *λ^k^* with *λ* > 1. Thus, the real values *L_i_*_,*t*_ and *L_t_* are the exponentially weighted losses of the *i*th expert and the predictor up to time *t*, respectively. We define *L*_0_ = *L_i_*_,0_ = 0 for simplicity. The weight of the expert is updated as
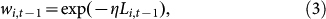
where *η* is a learning rate. Chernov and Zhdanov^16^ proposed a modified expert advice method in which they defined *a_k_*(*t*) = *ρ^t^*^−*k*−1^ and 0 < *ρ* < 1. We call this the “CZ method”.

The third step predicts future states of the target system by applying the obtained weighting to the future trajectories in the database^15^. We can generate a point prediction by simply adding the *q* steps ahead of the trajectories with the weights obtained in the second step as follows:
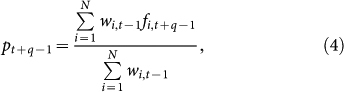
where *N* denotes the total number of experts. We can also generate a distributional prediction by assuming the distribution of observational errors, and summing this error distribution with the weights. To make these predictions online, we repeat the second and third steps iteratively.

### Upper bound of the regret of TEA

We derive an upper bound of the regret of TEA. The primary property peculiar to TEA lies in our definition of *a_k_*(*t*). We define the coefficient as *a_k_*(*t*) = *λ^k^*^−1^ or *λ^k^*, where *λ* > 1. The choice of these two options depends on each situation. When a given data is too short, we choose the latter, e.g. our prediction about the biomarker of prostate cancer, or PSA (prostate-specific antigen). Let 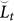 and 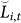 be the accumulated losses for the proposed method. We call these the *exponential accumulated losses* to distinguish them from the standard accumulated losses. In addition, we define the regret as 
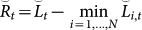
. The upper bound of the regret with *a_k_*(*t*) = *λ^k^*^−1^ is then given by
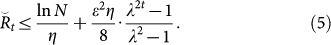


### Proof of the upper bound in our proposed method

We give a proof of Eq. (5) in a similar way to the Proof of Theorem 2.2 in Ref. 15. Define a new variable 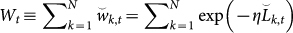. We will consider the upper and lower bounds of ln(*W_t_*/*W*_0_) to construct the upper bound of the regret. First, we obtain the lower bound of ln(*W_t_*/*W*_0_) as 
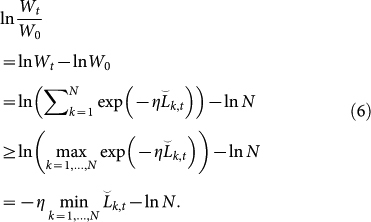


Second, we derive the upper bound of ln(*W_t_*/*W*_0_). Observe that 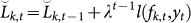. Then, we can reformulate ln(*W_t_*/*W_t_*_−1_) as follows:
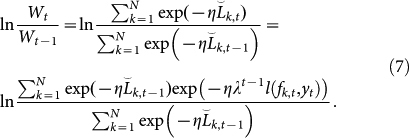


Equation (7) can be regarded as the average of random variable exp(−*ηλ^t^*^−1^*l*(*f_k_*_,*t*_, *y_t_*)) with a probability mass function proportional to 
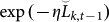
. Lemma 2.2 of Ref. 15 states that
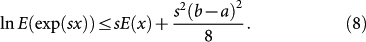
Here, we assume that *x* is a random variable satisfying *a* ≤ *x* ≤ *b*, and that the inequality holds when *s* is any real number.

Replace *s* by −*ηλ^t^*^−1^ and *x* by *l*(*f_k_*_,*t*_, *y_t_*). Then, the upper bound of Eq. (7) can be found using Eq. (8) as follows:
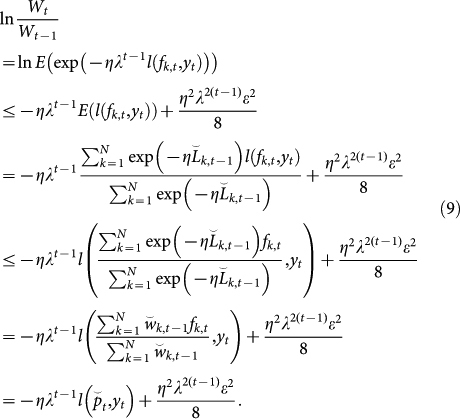
We assume that *l*(·,·) is convex as described above. Then, the upper bound of ln(*W_t_*/*W*_0_) can be derived as
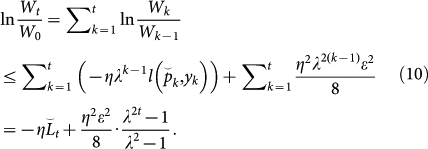
Because Eqs. (6) and (10) provide lower and upper bounds of ln(*W_t_*/*W*_0_), respectively, the following inequality is obtained:
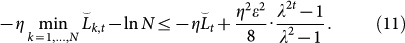
By substituting the regret 
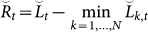
 into Eq. (11), we finally reach the following inequality:
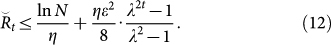


(Proof end)

### Optimization of the upper bound of TEA

We minimize the upper bound of Eq. (12) over *η*. First, we differentiate the upper bound with respect to *η* as follows:
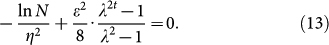


The solution is 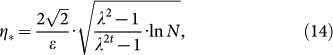
which gives the smallest upper bound. Replacing *η* in the upper bound of 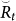 with η_*_, we obtain the following optimal upper bound 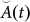:
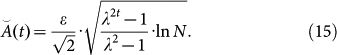
Although this optimal upper bound perhaps seems to be curious at a glance due to its exponential increase with *t*, this is caused by the definition of the accumulated losses Eqs. (1) and (2) with *a_k_*(*t*) = *λ^k^*^−1^_._ This regret can be compared with the normal types of regrets using relationship described in the next section. When *ε* = 1 and *λ* → 1, the optimal upper bound 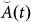 coincides with 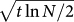, which is the upper bound obtained in the standard expert advice method^15^.

### Comparison between the proposed method and the Chernov–Zhdanov method

Here, we highlight the difference between the CZ method and the proposed TEA method. The first point of difference is the optimal upper bound of the regret. We briefly introduce the optimal upper bound of the CZ method^16^. Let 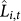 and 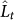 be the accumulated losses for the *i*th expert and the predictor for the CZ method, respectively. Then, the optimal upper bound 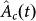 of the regret 
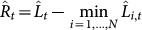
 for the CZ method is given by
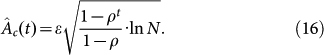
Note that we assume the case where the value of the decay rate *ρ* does not depend on *t* or *k*. See Ref. 16 for the proof.

Although we cannot directly compare these regrets, we can compare them after normalization. Assuming that the decay rates are equal, namely *λ* = *ρ*^−1^, the regrets 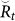 and 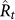 have the following relation:
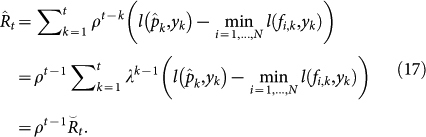
Using this relation, a comparison between the two upper bounds is feasible. Multiplying the optimal upper bound 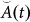 by *ρ^t^*^−1^, we obtain the normalized optimal upper bound 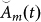 as
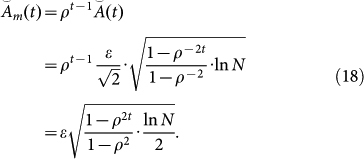


Then, the following relation is obtained:
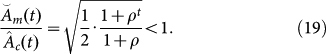
This result means that the normalized optimal upper bound of the proposed method is always smaller than that of the CZ method when 0 < *ρ* < 1.

Next, we compare the weights produced by the two methods. Let 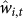 and 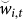 be the weights of the *i*th expert at time *t* in the CZ and TEA methods, respectively. Similarly to the derivation of Eq. (17), the accumulated losses of both methods are related by 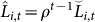. Substituting this relation into 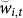, we have
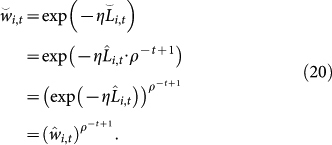
Equality (20) means that the proposed TEA method tends to assign reliable experts with heavier weights than the CZ method. This implies that 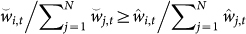 for reliable experts because *ρ*^−*t*+1^ ≥ 1.

### Examples of time-series prediction for mathematical models

We demonstrate the superiority of the TEA method to both the CZ method and the standard expert advice in online time-series prediction using toy examples. We use the Hénon map^17^ and the Ikeda map^18^ for our demonstration. These two models are commonly used to test nonlinear time-series analysis methods, which exhibit typical unstable chaotic dynamics. First, we generate time-series for the database using various values of parameters. We then generate a target time-series for prediction using a set of parameter values that is different from those used to generate the database. We prepare *M* × *S* experts for the database, where *M* is the number of parameter sets. For each parameter set, we generate *S* experts with different initial conditions. In numerical simulations of TEA, we set *ε* = 1 and 
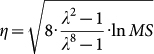
. We also set *λ* = *ρ*^−1^ and *ρ* = 0.9. See Algorithm 2 in Ref. 16 for the implementation of the CZ method, and Ref. 15 for that of the standard expert advice.

**Figure 2:**
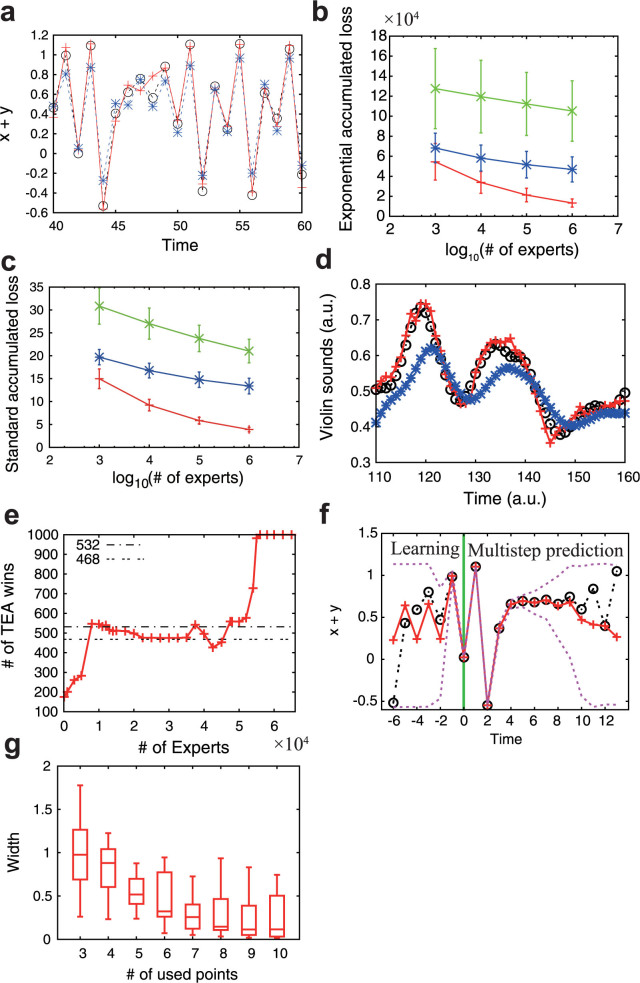
Examples of prediction by TEA. (a) Online point prediction of the Hénon map. (b, c) The number of experts versus the exponential accumulated loss of errors and the standard accumulated loss of errors in the prediction of the Hénon map. (d) Online point prediction of violin sounds. (e) The number of experts versus the number of wins out of 1,000 online prediction trials against the persistence prediction in the violin example. (f) Distribution prediction of the Hénon map. (g) Confidence interval width against the number of used points shown in box plots. In panels (a) and (f), actual observations are shown with black ○ and dotted lines, TEA predictions are shown with red + and solid lines, and the prediction given by the method of Chernov and Zhdanov is shown with blue * and dashed lines. In panel (f), the purple dotted lines show the 95% confidence interval of the distribution prediction, and the green line divides the learning period and the multistep prediction period. In panels (b) and (c), red, blue, and green error bars correspond to TEA, the method of Chernov and Zhdanov, and the standard expert advice, respectively. In panel (e), the dotted lines show the 95% confidence interval under the null-hypothesis of even chance.

The Hénon map^17^ is a two-dimensional map defined as 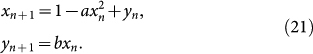
We set the parameters at *a* = 1.35 and *b* = 0.15 to generate the target time-series. Note that the dynamics produced by this parameter set is of deterministic chaos. The experts' parameters are uniformly chosen from *a* ∈ [1.3,1.4] and *b* ∈ [0.1,0.2]. The initial conditions *x*_0_ and *y*_0_ are randomly chosen in [−0.02, 0.02] × [−0.02, 0.02], and the map is iterated for 1,000 steps to eliminate transient effects. We assume that we observe and predict the value of *x* + *y*. We use this assumption because we can observe a scalar biomarker of PSA in the prostate cancer application discussed later. The results presented in Figs. 2a, 2b, and 2c show that the proposed TEA achieves better online time-series prediction than the standard expert advice and the CZ method. We choose *M* = 100 and *S* = 1,000 in Figs. 2a, 2f, and 2g. Another example of the Ikeda map is shown in Supplementary Fig. S1 (see also Supplementary Information).

The proposed TEA method provides the best online prediction in different toy examples. The more experts we use, the smaller the prediction errors become. When a large number of experts are used, the proposed TEA tends to achieve the best online time-series prediction. We need to decay the past information in these examples, because the unstable chaotic dynamics rapidly loses the memories.

### Examples of time-series prediction for real datasets

We now consider two real datasets: violin sounds^19^ and the membrane potential of squid giant axons^20^. The violin sounds are RWC-MDB-I-2001-W05 No. 15 in the RWC Music Database (Musical Instrument Sound). Previous studies on squid giant axons have demonstrated the chaotic nature of the underlying dynamics^20^,^21^,^22^,^23^. These time-series are both scalar and real-valued. We divide each time series into two. The first part is used to build the database, and the second constructs the targets for online prediction. We use *M* = 1,000 and *M* = 120 targets for the analysis of violin sounds and squid giant axon data, respectively. The lengths of the target data are 311 for the violin data and 51 for the squid giant axon data; numbers and lengths of target data are determined by the lengths of the original datasets.

**Figure 3:**
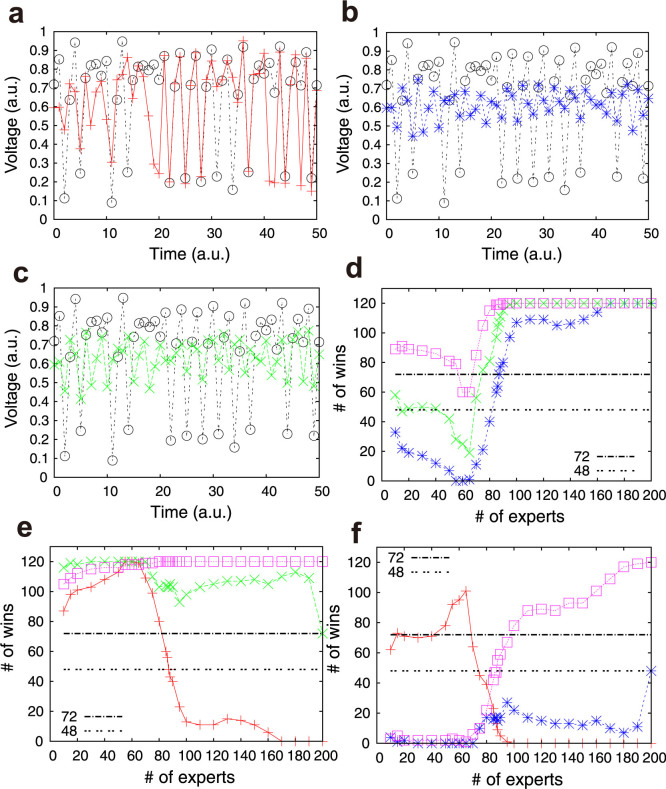
Online time-series prediction of membrane potential of squid giant axon. The decay parameter was fixed at *ρ* = 0.9. (a, b, c) The observed time-series is represented by black lines. The number of experts is 160. The predicted time-series represented by a red dashed line in (a), a blue dashed line in (b), and a green dashed line in (c) are obtained by our method, CZ, and the standard expert advice, respectively. (d) The number of times our method outperforms CZ (blue *), the standard expert advice (green ×), and the average prediction (purple □) (out of 120) are shown. When the number of wins for a method is greater than 71 (the dashed-dotted horizontal line), it is significantly better than the other method in terms of the binominal test. When it is smaller than 49 (the double-dotted line), the opposite is true. (e) The number of times the CZ method outperforms our method (red +), the standard expert advice (green ×), and the average prediction (purple □). (f) The number of times the standard expert advice outperforms our method (red +), CZ (blue *), and the average prediction (purple □).

We compare five methods using these real data. These are our TEA method, the CZ method, the standard expert advice, the persistence prediction, and the average prediction. The persistence prediction is a method that we let the current value to be the prediction for the next time point. We compare each pair of the method individually, and count the number of points at which the prediction by one method is better than the other for each target time-series. If one method is superior at more than half the data points, we declare that method the winner on the target data. We exclude the initial ten points from the analysis, because we cannot prepare the learning part. Finally, we count the number of wins and losses for each pair among the five methods. In the TEA numerical simulations, we set *ε* = 1 and 
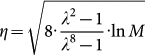
. We also set *λ* = *ρ*^−1^ and *ρ* = 0.9. The violin sound^19^ results are shown in Figs. 2d and 2e, and Tables 1. For this dataset, our method and the persistence prediction produce much better results than the other methods. Therefore, we next compare our TEA method with the persistence prediction with respect to the number of experts. We use the binominal test for the analysis, i.e., if the number of wins is greater (smaller) than 531 (469), the method is significantly superior (inferior) to the other method with respect to the 95% confidence level two-sided binominal test. When the number of experts is large, our TEA method is significantly superior to the persistence prediction, as shown in Fig. 2e and Table 1. In the example of squid giant axon^20^, the proposed TEA is also better than the other four methods when the number of experts is large, especially when greater than or equal to 87, as shown in Fig. 3 and Table 1.

In conclusion, our TEA method tends to provide the best prediction when the number of experts is large. The precise number of experts for which this is the case may change depending on the given data, the length of targets, and the decay parameter.

### Distribution prediction to the mathematical models

We applied the distribution prediction to time-series of the Hénon map. The distribution prediction will be explained in the later Method section. The setup is similar to that for the point prediction, except that we provide the prediction as a distribution. The results are presented in Figs. 2f and 2g. The width of the distribution prediction is narrow immediately after the learning period (Fig. 2f), then grows gradually as the number of prediction steps increases because of the instability of the underlying dynamics. The predicted confidence interval tends to contain the actual values. When we increase the number of points used for prediction, the width of the distribution prediction becomes narrower (Fig. 2g). We use values of *S* = 1,000 and *M* = 100 in Figs. 2f and 2g. In the TEA numerical simulations, we set *ε* = 1 and 
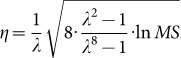
. The number of trials is 40 in each box in Fig. 2g. Restricting the range of *λ* to 1 < *λ* < 2 gives a better prediction. We generate the target and experts' time-series as in the previous section. We also obtain the distribution prediction of the Ikeda map using *S* = 1,000 and *M* = 100, as shown in Fig. S1f. The result is very similar to that of the Hénon map. Again, restricting the range of *λ* to 1 < *λ* < 2 gives a better prediction.

### Mathematical models of prostate cancer

TEA can be applied to clinical problems, such as the prediction of prostate-specific antigen (PSA) after some initial treatments, while waiting to start an additional treatment. We apply TEA to the prediction of tumor markers for prostate cancer PSA. Before the technical details, we introduce a mathematical model of prostate cancers in this section.

Patients had already received radical prostatectomy as an initial treatment. Then, clinicians followed postoperative PSA levels to determine when to commence salvage treatment. Although the timing at which patients start salvage treatment is an important problem, there is no definitive agreement on when this to be started. Currently, clinicians are determining the start of salvage treatment based on their discretion. The clinical part of this study was approved by the ethics committees of Jikei University School of Medicine and The University of Tokyo. All patients provided written informed consent. Cancer cells tend to thrive under an androgen-rich environment. Meanwhile, lowering androgen levels makes cancer cells grow slowly or rather decline. Because of this characteristics, clinicians suppress the androgen concentration with hormone therapy. However, when cancer cells remain exposed to an androgen-poor environment, they often acquire the ability to grow without androgen. This growth signals a cancer relapse. Intermittent androgen suppression was proposed to delay the relapse of cancer^24^. In intermittent androgen suppression, we start hormone therapy, but stop when PSA levels have decreased sufficiently. Then, we wait until PSA increases and reaches a threshold value. After reaching this threshold, we resume hormone therapy. We repeat this process to delay the relapse. However, clinical trials show that the effects of intermittent androgen suppression depend on individual patients, and are limited^25^,^26^.

Here, we use a mathematical model^8^ of intermittent androgen suppression for prostate cancer^24^,^25^,^26^. This model was constructed based on data of Canadian patients^25^,^26^ whose PSA had increased to some extent after radiation therapy, and were later treated by intermittent androgen suppression. Because the model of Ref. 8 has a small number of parameters, it is reasonable to predict the future PSA values with this simple model and very short time-series, although several mathematical models have been proposed to describe dynamics under intermittent androgen suppression^4^,^5^,^6^,^8^,^10^,^27^,^28^,^29^,^30^,^31^. In the model described in Ref. 8, we assume that there are three classes of cancer cells: androgen dependent cancer cells *x*_1_, androgen independent cancer cells generated through reversible changes *x*_2_, and androgen independent cancer cells generated through irreversible changes *x*_3_. When the hormone therapy is underway, *x*_1_ may change to *x*_2_ or *x*_3_. When the hormone therapy is stopped, *x*_2_ may return to *x*_1_, whereas *x*_3_ cannot return to *x*_1_ or *x*_2_ because of genetic mutation. We previously verified two important properties of this model: namely, a piecewise linear model is sufficient to describe the dynamics of PSA, and the androgen concentration need not be explicitly included in the model^8^. Based on these verified properties, we can simply construct the mathematical model as 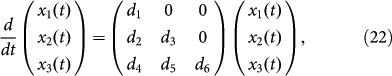
for the on-treatment period, and
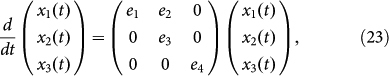
for the off-treatment period^8^. Here, *d*_1_, *d*_2_, *d*_3_, *d*_4_, *d*_5_, *d*_6_, *e*_1_, *e*_2_, *e*_3_, and *e*_4_ are model parameters. We assume that a PSA measurement is represented by *x*_1_ + *x*_2_ + *x*_3_ for simplicity. Thus, we must specify these 10 parameters for the dynamics and three other parameters for the initial conditions of *x*_1_, *x*_2_, and *x*_3_. If we try to find these 13 parameters directly only from a single target patient, we would need to obtain a long time-series. The application of the proposed TEA algorithm makes the required observation period of PSA measurements shorter by integrating observations from the target patient with the long time-series data of PSA measurements obtained from previous prostate cancer patients. We note that we only analyze the off-treatment period in this paper, because the target dataset is about the follow-up period after an initial treatment. Therefore, we need 4 control parameters and initial conditions.

### Construction of experts for prediction of PSA for prostate cancer

In this paper, we have two datasets; one is a dataset of Canadian patients with many data points; the other is a dataset of Japanese patients with short time points. We need a long time-series to efficiently estimate model parameters. Therefore, we select Canadian datasets for estimation of parameters and Japanese datasets for predicting targets.

In applying TEA to prostate cancer, we first prepared 72 sets of model parameters, each of which was obtained from one of 72 Canadian prostate cancer patients treated with intermittent androgen suppression. These parameters were obtained from Ref. 8. We note that our prediction target dataset corresponds to the off-treatment period in the model^8^. Second, we chose the number of observation points to use as known data points. This must be at least three because of the model dimensions^8^. Third, using each set of parameters, we determined the initial model state to minimize the fitting error between the initial three or more PSA measurements and the model output. The optimal initial conditions were selected by minimizing the following cost function:
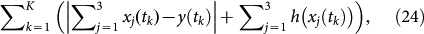
where *h*(*x*) = 10^15^(1 − *x*) for *x* < 0 and *h*(*x*) = 0 for *x* ≥ 0, where *t_k_* is the *k*th observation time. We denote the number of observation points used for learning by *K*. The method of obtaining the initial conditions was similar to that in Ref. 8. Fourth, we ran the model with each set of parameters and the corresponding initial conditions to construct the database of experts *f_i_*_,*t*_; thus, we have 72 experts.

### Estimation of learning parameters

We applied the second step of the TEA algorithm to determine the weights of the PSA measurements. Then, we applied the third step of the TEA algorithm to obtain the distribution prediction. We determined the optimal decay rate *λ* by minimizing the error between the last learning observation and the prediction. We restricted the range of *λ* to 1 < *λ* < 2 to obtain better predictions. The standard deviation *σ* is estimated as follows. We ran the distribution prediction with the obtained initial conditions and the decay parameter. We set the standard deviation *σ* to the mean of the absolute errors between the median of the distribution prediction and the corresponding observation when the mean is taken during the learning period.

### Application of TEA to prediction of PSA for prostate cancer

**Figure 4:**
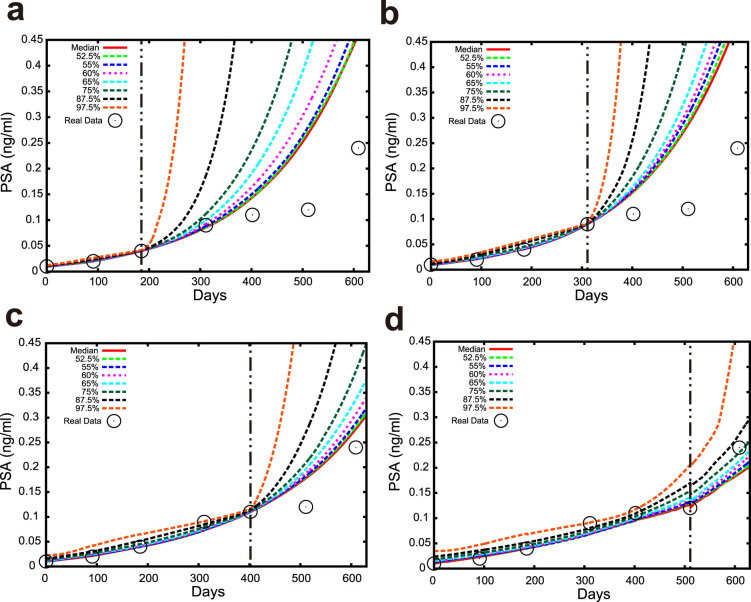
Results of the distribution prediction of PSA in a patient. The black ○ shows the actual PSA observations. We used the (a) first three, (b) four, (c) five, and (d) six observation points to predict the next data points for the patient with the TEA(*v* = 1). The red curve shows the median distribution prediction. Other dotted lines indicate as denoted in each figure. The same patient data are used in all the figures.

**Figure 5:**
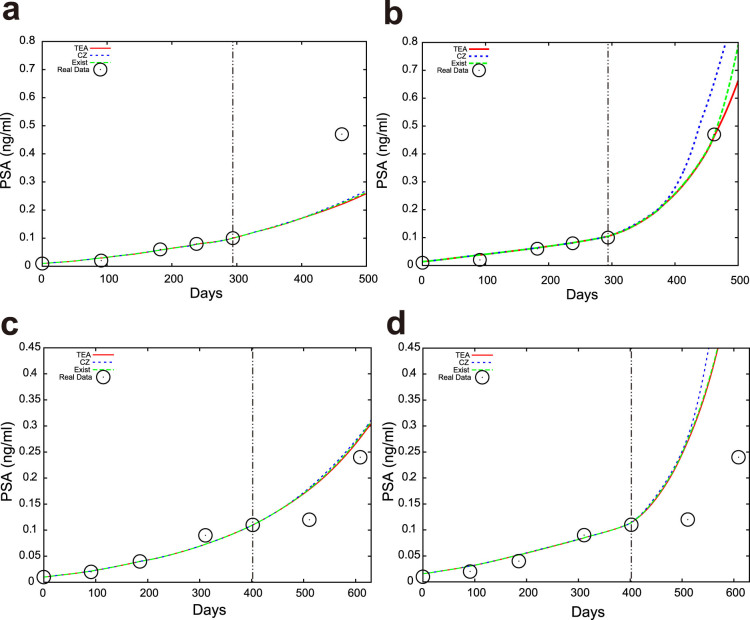
Examples of PSA predictions using TEA (red), CZ (blue), and the standard expert advice (green). The black ○ shows the actual PSA observations. The red, blue, and green curves were obtained from TEA, the Chernov-Zhdanov method, and the standard expert advice, respectively. We show the median in (a) and (c). We chose 87.5% points of the predicted distributions in (b) and (d). The same patient data are used in (a) and (b). Other patient data are used in (c) and (d).

We predict the values of PSA with distribution prediction. The distribution prediction of PSA with TEA is shown in Fig. 4. Here we evaluate the larger side of the predicted distribution, because overlooking high PSA is highly undesirable in a real clinical setting. We show seven points *u_t_*(*Q*) of the predicted distribution (97.5%, 87.5%, 75%, 65%, 60%, 55%, and 52.5%) in these figures, where *u_t_*(*Q*) is defined as
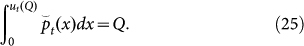
Note that *Q* is the intended value of the probability, i.e. 0.975, 0.875, 0.75, 0.65, 0.6, 0.55, and 0.525, respectively, in this situation. We obtained the proportion of PSA values that are less than the intended probability for each *Q*, and counted the PSA data points that are next to the final data points in the learning period; namely, if we are using three data points for learning, we count the fourth data point. In this paper, we focus on the predictability of the next point. The results are summarized in Table 2. Note that TEA can predict not only the next data point, but also those far in the future. We predicted the future PSA values for 88, 86, 80, and 69 patients when we used the first three, four, five and six time points, respectively. We also conducted numerical simulations using CZ and the standard expert advice. The predicted distributions were different for each method, as shown in Fig. 5. In numerical simulations, we set *ε* = 1. We arrange the learning rate as four constant values 
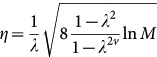
 with *v* = 1, 2, 3, and 4. In addition, we increase *v* as the number of the learning points increases. We note that *M* = 72 is the number of experts. We also arrange the learning rate as 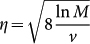 for the standard expert advice and 
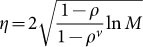
 for the CZ method.

TEA exhibits the best performance among the three methods, because each proportion tends to be closest to the specified value of *Q*. These results imply that our proposed prediction method may be reasonable for real applications in a clinical setting. We also checked the prediction performance in terms of the median using the mean absolute error (MAE) as summarized in Supplementary Table S1. As a result, TEA shows the best performance in the meaning of the average MAE among the four cases. We note that the CZ method showed good performance in terms of the root mean square error (RMSE), however, we believe that the MAE suits our situation because we employed the absolute error function for the learning period.

## Discussion

In general, clinicians provide a salvage treatment with patients who had recurrence after surgery. Although many studies show the clinical benefit of a salvage treatment for patients with prostate cancer, current studies have reported that an earlier salvage treatment, especially for local recurrence, could improve clinical outcomes^32^. These results suggest that post-operative patients with lower PSA values may have a higher frequency of local recurrence that could be efficiently treated by radiotherapy. If clinicians can accurately assess the PSA failure at an earlier stage than the present standard criterion of PSA failure which is that the PSA value increases to 0.2 (ng/ml) or more after surgery, salvage treatments could be more effectively scheduled for each patient, improving the final clinical outcome^33^. However, there is still no standardized criterion to determine the best timing of salvage treatments^32^,^33^. Combined with a mathematical model^8^, TEA or its further extensions may be able to potentially predict the PSA dynamics in patients before PSA failure. Therefore, the proposed TEA could become the basis of a new standard index for earlier prediction of PSA failure using a simple mathematical solution, that offers important information for a suitable salvage treatment after surgery^7^,^34^,^35^.

The more experts we use, the more (numerically) accurate the prediction tends to become (Figs. 2b, 2c, and 2e); in this sense, the accumulation of datasets is important. Additionally, the longer the learning period, the more accurate the TEA prediction tends to become in the toy examples (Fig. 2g). This could be because the toy examples have bounded unstable dynamics. The prediction error does not monotonically decrease with an increase of the learning data points in the example of prostate cancer (Tables 2 and S1), because PSA tends to increase monotonously in time. TEA exhibits the best performance in our analyses. The proposed combination of the expert advice with a predicted distribution enhances the reliability of prediction. This is important in many applications, and especially in medicine.

In summary, we have demonstrated that TEA can infer the state of a target system, by combining its short time-series and the expert advice constructed as a collection of longer time-series. The proposed TEA may be applied to any problems where a short time-series and its database are given, as demonstrated in the violin and squid giant axon examples, although we primarily intend to apply TEA in clinical settings, such as inferring the state of a disease using a short time-series from the target patient and longer time-series from previous patients with the same disease. We hope that TEA improves the overall survival and/or quality of life for patients.

## Methods

### Standard expert advice method

The expert advice method^15^ is an online predictor in machine learning. We briefly introduce the standard expert advice method in this section. See the book of Cesa-Bianchi and Lugosi^15^ for a more detailed introduction. The expert advice consists of experts and a predictor. At each time step, each expert gives the prediction on the future. The predictor makes a prediction for the future by weighting these pieces of advice based on the experts' prediction history. After a new outcome is observed, the predictor updates the experts' weights using the losses produced in the current step. We iterate these steps to realize online prediction. Let *f_i_*_,*t*_ be the *i*th expert's advice at time *t* and *N* be the number of experts. We assign each expert the weight *w_i_*_,*t*_ at time *t*, and obtain the prediction by averaging the experts' advice as
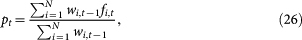

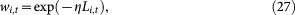
where *p_t_* is the prediction at time *t*, *η* is a constant, and *L_i_*_,*t*_ is the accumulated loss for the *i*th expert at time *t*. Better experts have smaller accumulated losses, and hence have larger weights. The accumulated losses for the *i*th expert and the predictor at time *t* are
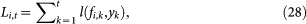

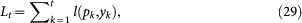
where *y_k_* is the observation at time *k*, and *l*(*x*, *y*) is a convex loss function, typically the absolute error |*x* − *y*| or squared error (*x* − *y*)^2^. We evaluate the performance of the predictor by a regret, which is defined as the predictor's accumulated loss minus the accumulated loss for the best expert. Mathematically, the regret *R_t_* is defined as 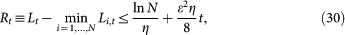
where *ε* is the maximum value of |*l*(·,·)|. Namely, the regret is bounded above by the right-hand side of Eq. (30) (see Ref. 15 for the derivation). We obtain the following optimal constant η_*_ by minimizing the upper bound over *η*:
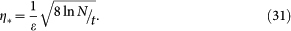
When replacing *η* with η_*_, we obtain the optimal upper bound of the regret *R_t_* as 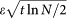. We call the accumulated losses defined in Eqs. (28) and (29) the *standard accumulated losses*.

Although the standard expert advice can be applied in many cases, the method is not suited to the prediction of unstable systems, in which the recent history should be emphasized to predict the future more accurately. Thus, we extended the standard expert advice by placing greater weights on recent past information. We call our extension the temporal expert advice, or TEA.

### Distribution prediction

Here, we extend the TEA method for point prediction to the prediction of distribution, so that we can handle the prediction of biomarkers. For this purpose, we introduce the distribution prediction of the *i*th expert at time *t* 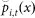 as follows:
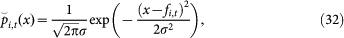
where *σ* is the standard deviation. This distribution is given under the assumption that a point prediction *f_i_*_,*t*_ is disturbed by various errors and that the error is normally distributed around the point prediction. Then, the predictor for the distribution prediction is given by
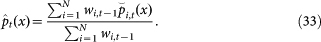
We determine the optimal decay rate *λ* by minimizing the absolute error between the final learning point and the corresponding observation point. The standard deviation *σ* is set to be the absolute difference between the point prediction 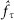 and the observation 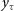 at the final learning point under the estimated *λ* above as 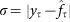, where 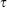 is the number of the learning points. We note that we determined these parameters with a modified way in the prediction of PSA because of its instability.

## Supplementary Material

Supplementary Information Supplementary Information

## Figures and Tables

**Table 1:** Analysis of violin sounds and the membrane potential of squid giant axon. The number of wins between each pair of the five prediction methods is shown. The each number indicates the number of experts in each case.

Method	Method of Comparison									
#	100 (Violin sounds)					1000 (Violin sounds)				
	TEA	CZ	Exist	Persistence	Average	TEA	CZ	Exist	Persistence	Average
TEA	—	1000	509	175	1000	—	1000	785	201	1000
CZ	0	—	0	0	1000	0	—	21	0	1000
Exist	491	1000	—	19	1000	215	979	—	79	1000
Persistence	825	1000	981	—	1000	799	1000	921	—	1000
Average	0	0	0	0	—	0	0	0	0	—
#	30000 (Violin sounds)					55000 (Violin sounds)				
	TEA	CZ	Exist	Persistence	Average	TEA	CZ	Exist	Persistence	Average
TEA	—	1000	1000	475	1000	—	1000	998	982	1000
CZ	0	—	0	0	1000	0	—	0	0	1000
Exist	0	1000	—	0	1000	2	1000	—	2	1000
Persistence	525	1000	1000	—	1000	18	1000	998	—	1000
Average	0	0	0	0	—	0	0	0	0	—
#	30 (Squid axon)					90 (Squid axon)				
	TEA	CZ	Exist	Persistence	Average	TEA	CZ	Exist	Persistence	Average
TEA	—	17	50	120	88	—	80	115	120	120
CZ	103	—	120	120	115	40	—	103	120	120
Exist	70	0	—	120	2	5	17	—	120	59
Persistence	0	0	0	—	0	0	0	0	—	0
Average	32	5	118	120	—	0	0	61	120	—
#	120 (Squid axon)					180 (Squid axon)				
	TEA	CZ	Exist	Persistence	Average	TEA	CZ	Exist	Persistence	Average
TEA	—	109	120	120	120	—	120	120	120	120
CZ	11	—	105	120	120	0	—	113	120	120
Exist	0	15	—	120	89	0	7	—	120	117
Persistence	0	0	0	—	0	0	0	0	—	0
Average	0	0	31	120	—	0	0	3	120	—

**Table 2:** Prediction results for real PSA datasets. The proportions of PSA data points that are followed by the TEA, CZ, and Existing prediction are shown against *Q*%, points of the predicted distribution from below. A learning rate *η* is changed with *v*. An abbreviation t.v. indicates time-varying. We consider data points that are next to the final point used for the learning period. We underline the best method in each case.

CI *Q*	Used points	TEA					CZ					Exist				
		t.v.	*v* = 1	2	3	4	t.v.	1	2	3	4	t.v.	1	2	3	4
**97.5**	3	**100**	**100**	**100**	**100**	**100**	**100**	**100**	**100**	**100**	**100**	**100**	**100**	**100**	**100**	**100**
	4	100	**98.8**	100	100	100	100	100	100	100	100	100	100	100	100	100
	5	**100**	**100**	**100**	**100**	**100**	**100**	**100**	**100**	**100**	**100**	**100**	**100**	**100**	**100**	**100**
	6	**100**	92.8	**100**	**100**	**100**	**100**	**100**	**100**	**100**	**100**	**100**	**100**	**100**	**100**	**100**
**87.5**	3	**89.8**	92	90.9	**89.8**	**89.8**	**89.8**	**89.8**	**89.8**	**89.8**	**89.8**	89.8	92	**89.8**	**89.8**	**89.8**
	4	**93**	94.2	94.2	**93**	**93**	**93**	**93**	94.2	**93**	**93**	93	94.2	94.2	**93**	**93**
	5	98.8	**96.2**	97.5	98.8	**96.2**	98.8	98.8	98.8	98.8	98.8	98.8	97.5	97.5	98.8	98.8
	6	98.6	**87**	91.3	97.1	97.1	98.6	98.6	98.6	98.6	98.6	98.6	95.7	95.7	97.1	97.1
**75**	3	**75**	**75**	**75**	**75**	**75**	**75**	**75**	**75**	**75**	**75**	**75**	**75**	**75**	**75**	**75**
	4	**84.9**	**84.9**	**84.9**	**84.9**	**84.9**	**84.9**	**84.9**	**84.9**	**84.9**	**84.9**	**84.9**	**84.9**	**84.9**	**84.9**	**84.9**
	5	91.2	**87.5**	90	90	90	91.2	91.2	91.2	91.2	91.2	90	90	90	90	90
	6	89.9	**75.4**	78.3	84.1	85.5	91.3	89.9	89.9	89.9	91.3	87	87	87	87	87
**65**	3	**69.3**	**69.3**	**69.3**	**69.3**	**69.3**	**69.3**	**69.3**	**69.3**	**69.3**	**69.3**	**69.3**	**69.3**	**69.3**	**69.3**	**69.3**
	4	**79.1**	80.2	80.2	80.2	80.2	**79.1**	**79.1**	**79.1**	**79.1**	**79.1**	80.2	80.2	80.2	80.2	80.2
	5	88.8	**82.5**	87.5	88.8	88.8	88.8	88.8	88.8	88.8	88.8	88.8	87.5	87.5	88.8	88.8
	6	84.1	**72.5**	**72.5**	76.8	78.3	82.6	82.6	84.1	84.1	84.1	84.1	78.3	79.7	81.2	81.2
**60**	3	**67**	68.2	68.2	**67**	**67**	**67**	**67**	**67**	**67**	**67**	**67**	68.2	**67**	**67**	**67**
	4	79.1	**77.9**	79.1	79.1	80.2	**77.9**	**77.9**	**77.9**	**77.9**	**77.9**	80.2	79.1	79.1	79.1	79.1
	5	85	**81.2**	83.8	85	85	86.2	85	85	86.2	86.2	85	83.8	85	85	85
	6	78.3	**71**	72.5	75.4	76.8	79.7	78.3	78.3	79.7	79.7	78.3	75.4	76.8	78.3	78.3
**55**	3	65.9	**64.8**	**64.8**	**64.8**	65.9	65.9	65.9	65.9	65.9	65.9	65.9	**64.8**	**64.8**	**64.8**	65.9
	4	**73.3**	**73.3**	**73.3**	**73.3**	74.4	**73.3**	74.4	**73.3**	**73.3**	**73.3**	**73.3**	74.4	74.4	74.4	74.4
	5	82.5	**78.8**	**78.8**	82.5	82.5	81.2	82.5	82.5	82.5	82.5	82.5	81.2	81.2	82.5	82.5
	6	75.4	**71**	72.5	72.5	73.9	76.8	72.5	73.9	73.9	75.4	75.4	75.4	73.9	73.9	73.9
**52.5**	3	**64.8**	**64.8**	**64.8**	**64.8**	**64.8**	**64.8**	**64.8**	**64.8**	**64.8**	**64.8**	**64.8**	**64.8**	**64.8**	**64.8**	**64.8**
	4	70.9	73.3	72.1	70.9	**69.8**	70.9	70.9	70.9	70.9	70.9	72.1	74.4	73.3	72.1	72.1
	5	80	**76.2**	77.5	80	80	78.8	80	80	80	78.8	80	78.8	80	80	80
	6	72.5	**69.6**	**69.6**	71	72.5	75.4	71	72.5	72.5	73.9	72.5	73.9	72.5	72.5	73.9
