# Supplementary material for: Predicting disease progression from short biomarker series using expert advice algorithm
Source: Sci Rep. 2015 May 20;5:8953. doi: 10.1038/srep08953 (PMC5386184; doi:10.1038/srep08953)
Supplement: Supplementary Information [file srep08953-s1.pdf]

Supplementary Information of "Predicting disease progression from short biomarker series using expert advice algorithm"

Authors:

Kai Morino[1]*, Yoshito Hirata[1,2], Ryota Tomioka[3], Hisashi Kashima[4], Kenji Yamanishi[1,5], Norihiro Hayashi[6], Shin Egawa[6], Kazuyuki Aihara[1,2]

Affiliations:

[1]Graduate School of Information Science and Technology, The University of Tokyo, Tokyo 153-8505, Japan.

[2]Institute of Industrial Science, The University of Tokyo, Tokyo 153-8505, Japan.

[3]Toyota Technological Institute at Chicago, Chicago, Illinois 60637, USA.

[4]Graduate School of Informatics, Kyoto University, Kyoto 606-8501, Japan.

[5]CREST, JST, Honcho, Kawaguchi, Saitama 332-0012, Japan.

[6]Department of Urology, Jikei University School of Medicine, Tokyo 105-8461, Japan.

*To whom correspondence should be addressed. E-mail: morino@mist.i.u-tokyo.ac.jp

Supplementary Methods:

Tables of contents

1. Prediction for the Ikeda map
2. Evaluation of the median prediction
3. Supplementary Table S1
4. Supplementary Figure S1

<u>1. Prediction for the Ikeda map</u>

The Ikeda map[18] is a two-dimensional map, and is defined as

$$\begin{aligned} x_{n+1} &= 1 + a(x_n \cos\theta - y_n \sin\theta), \\ y_{n+1} &= a(x_n \sin\theta + y_n \cos\theta), \\ \theta &= 0.4 - \frac{b}{1 + x_n^2 + y_n^2}. \end{aligned} \qquad \text{(S1)}$$

We choose $a = 0.8$ and $b = 9$ for the target time-series. The underlying dynamics is chaotic with this parameter set. The experts' parameters are chosen uniformly from $a \in [0.7, 0.9]$ and $b \in [8.5, 9.5]$, and the initial conditions x_0 and y_0 are both

randomly selected from $[-0.05, 0.05] \times [-0.05, 0.05]$. We iterate the map for 1,000 steps to overcome any initial transient effects. We presume that we observe and predict $x + y$. The predictions shown in Fig. S1 demonstrate that the proposed TEA method yields smaller standard and exponential accumulated losses than the standard expert advice method and the CZ method. We choose M = 100 and S = 1,000 in Fig. S1**a**, S1**b**, and S1**c**.

2. Evaluation of the median prediction

In the main text, we obtain the distribution prediction of PSA. Here, we evaluate the accuracy of the prediction using the median. We estimate the accuracy in terms of the root mean square error (RMSE) and the mean absolute error (MAE). Let us denote the observation value of the *i*th patient by y_i and predictions (median) of the *i*th patient by $\hat{y}_i$. The RMSE is given as

$$RMSE = \sqrt{\frac{1}{N}\sum_{i=1}^{N}\left(y_i - \hat{y}_i\right)^2}, \qquad \text{(S2)}$$

where N is the number of patients. The MAE is given as

$$MAE = \frac{1}{N}\sum_{i=1}^{N}\left|y_i - \hat{y}_i\right|. \qquad \text{(S3)}$$

We obtain the RMSE and the MAE for the prediction of PSA as shown in Supplementary Table S1. TEA shows the best performance in terms of the MAE, however, the CZ method shows the best performance in terms of the RMSE. In the main text, our learning algorithm used an absolute error function in its learning period. Therefore, we believe that we should evaluate the performance with respect to the MAE.

Supplementary Table S1: The RMSE and the MAE of the median prediction of PSA. We underline the best performance in each case. Abbreviations t.p and t.v. mean time points and time-varying, respectively.

		RMSE					MAE				
		3 t.p.	4 t.p.	5 t.p.	6 t.p.	Ave.	3 t.p.	4 t.p.	5 t.p.	6 t.p.	Ave.
TEA	t.v.	0.139	0.193	0.173	0.265	0.193	0.0698	0.0901	0.0946	0.108	0.0908
	$v = 1$	0.137	0.219	0.202	0.592	0.288	0.07	0.0961	0.0937	0.162	0.106
	$v = 2$	0.138	0.201	0.208	0.26	0.202	0.07	0.0916	0.0975	**0.0947**	0.0884
	$v = 3$	0.139	0.201	0.202	0.263	0.201	0.0698	0.0916	0.0977	0.0995	0.0896
	$v = 4$	0.139	0.188	0.19	0.229	0.186	0.0699	0.0868	0.0982	0.0977	**0.0881**
CZ	t.v.	0.139	0.201	0.166	0.224	0.183	0.0698	0.0934	0.0934	0.104	0.0902
	$v = 1$	0.139	0.192	0.205	0.265	0.2	0.07	0.0894	0.102	0.108	0.0922
	$v = 2$	0.139	0.199	0.174	0.248	0.19	0.0698	0.0925	0.0954	0.108	0.0915
	$v = 3$	0.14	0.2	**0.162**	0.221	0.181	0.0697	0.0932	**0.0915**	0.105	0.0897
	$v = 4$	0.14	0.202	0.164	**0.216**	**0.180**	0.0697	0.0935	0.0927	0.104	0.0899
Exist	t.v.	0.138	**0.187**	0.174	0.262	0.19	0.0696	**0.0852**	0.094	0.106	0.0888
	$v = 1$	**0.137**	0.219	0.209	0.264	0.207	**0.0694**	0.0952	0.0995	0.0984	0.0906
	$v = 2$	0.138	0.202	0.208	0.265	0.203	0.0698	0.0915	0.101	0.099	0.0902
	$v = 3$	0.138	0.202	0.205	0.268	0.203	0.0697	0.0918	0.101	0.104	0.0915
	$v = 4$	0.138	0.196	0.193	0.268	0.199	0.0696	0.0909	0.0984	0.105	0.091

Figure Legends S1: **Numerical simulations for the Ikeda map.** Analyses of time-series generated by the Ikeda map are shown. We set $a = 0.8$ and $b = 9$ for the target. The decay parameter was set to $\rho = 0.9$. **a, b, c** Time-series generated by the Ikeda map are represented by ○ with a black line. The predicted time-series represented by + with a red dashed line in **a**, * with a blue dashed line in **b**, and × with a green dashed line in **c** are obtained by our method, the CZ method, and the standard expert advice, respectively. **d, e** The dependence of accumulated errors L_t and $\breve{L}_t$ on the number of experts at time $t = 100$ is shown in **d** and **e**, respectively. The red, blue, and green lines and error bars show the mean and one standard deviation of **a**, **b**, and **c**, respectively. **f** Distribution prediction of the Ikeda map.

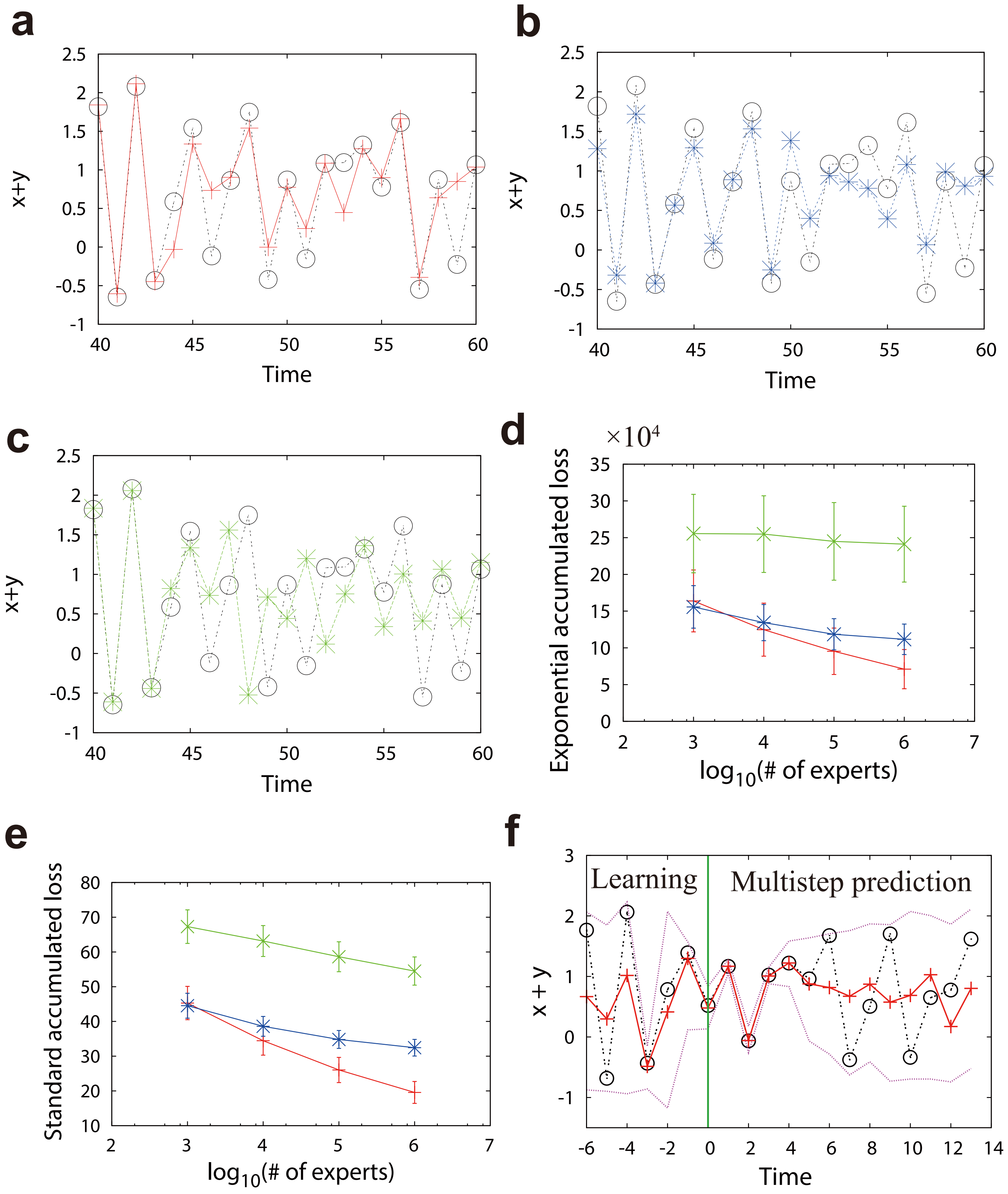

Supplementary Figure S1
